# Bedside Continuous Irrigation and Drainage as an Interim Local Treatment for Septic Arthritis of the Knee in the Medically Unstable Patient: A Case Report

**DOI:** 10.5704/MOJ.1507.003

**Published:** 2015-07

**Authors:** SS Khoo, KW Loi, KT Tan, AR Suhaeb, S Simmrat

**Affiliations:** Department of Orthopaedic Surgery, University Malaya, Kuala Lumpur, Malaysia

**Keywords:** Septic arthritis, knee, therapeutic irrigation, critically ill

## Abstract

Septic arthritis is a surgical emergency. Prompt diagnosis and immediate treatment reduce the destruction of articular cartilage and give better outcome. We describe a simple, minimally invasive closed tube irrigation system for the initial treatment of septic arthritis of the knee in a patient with complex medical problems who was unfit to undergo surgery.

## Introduction

It is a commonly known adage that septic arthritis is a surgical emergency, with the knee being the most commonly involved joint in adults^1,2^. Prompt diagnosis and immediate treatment reduce the destruction of cartilage thus give a generally better outcome^1,2^. Established treatments have moved on from open arthrotomy washout with subtotal synovectomy and joint immobilisation to be in favour of arthroscopic surgery, preservation of synovial tissue and early passive movement^2,3^. There is controversy regarding which method is superior. Regardless, the aim is to decompress the septic joint, reduce the bacterial load by mechanical irrigation and remove fibrin coatings, necrotic tissue and debris at the soonest^2^. This is coupled with parenteral antibiotics to achieve full functional recovery^1^.

## Case Report

A 52-year-old man with underlying human immunodeficiency virus (HIV) infection presented with a painful, swollen right knee and fever of two days duration. Examination revealed a tender, warm knee with gross effusion and limited range of motion. Initial aspiration produced 60 mL of pus and confirmed the diagnosis of right knee septic arthritis. The specimen was sent for bacteriologic, tuberculosis and fungal culture and sensitivity test.

We planned for emergency arthrotomy washout and debridement of the septic joint but were unable to proceed as he developed acute myocardial infraction upon admission. In view of the acute turn of events which rendered him temporarily unfit for surgical intervention, we performed bedside continuous irrigation and drainage of the septic knee whilst he received medical therapy for his heart condition. Meanwhile, he was empirically started on intravenous cloxacillin one gram six hourly.

**Fig. 1:**
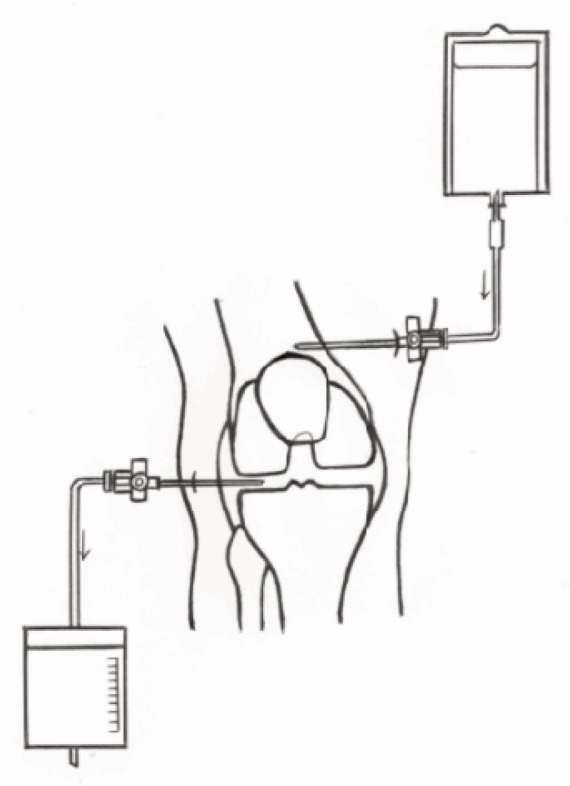
Closed tube irrigation system – positioning of the input and output channels.

**Fig. 2:**
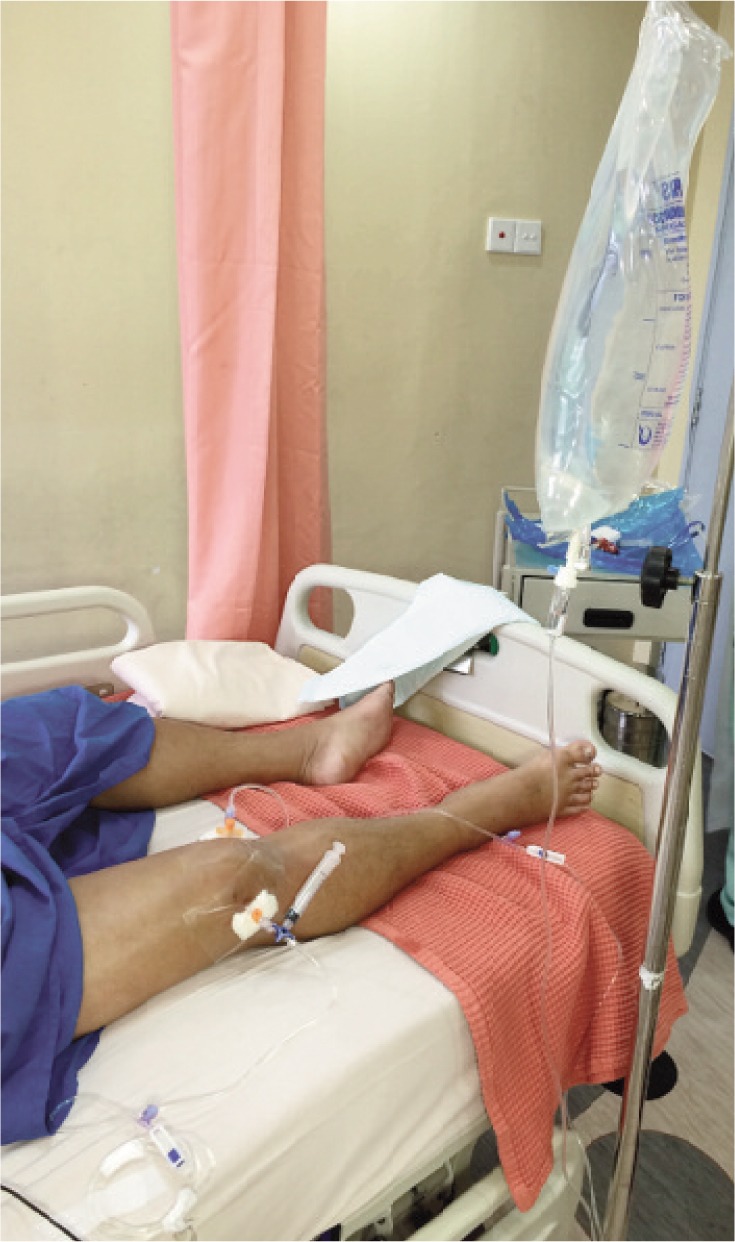
Closed tube irrigation system – clinical set-up.

We used two 14 gauge (orange) cannula; one as inflow inserted to the suprapatellar recess and the other as outflow inserted anterolaterally to the knee joint (Figure 1). The inflow cannula was connected via a standard drip line to a bag of physiological saline solution (sodium chloride 0.9%) placed on a drip stand at a height of 1.8 metres (Figure 2). The outflow cannula was attached via a three-way stopcock and drip line to a standard urine drainage bag hung at the side of the bed. We performed once a day intermittent saline distension and drainage for thirty minutes whilst maintaining continuous instillation and drainage by gravity at other times for a total of four days. We monitored for signs of leakage at every shift and kept a meticulous input and output chart.

The outflow irrigation fluid became clear after twenty four hours of continuous irrigation. The irrigation system was removed after four days when clinical improvement was seen, as evidenced by absence of pain, resolution of fever and decreased infective parameters (white cell count from 13.4 to 9.7 × 109/L, C-reactive protein level from 47.6 to 6.3 mg/dL and erythrocyte sedimentation rate unchanged 50 to 52 mm/hr). Culture of both blood and knee aspirate revealed Streptococcus pyogenes which was sensitive to penicillin, for which he received intravenous aqueous crystalline penicillin G 2.4 mega units six hourly for six weeks. The patient was advised for a formal washout and debridement when he was more stable but he was reluctant due to high anaesthetic risk. He also did not return for follow up upon discharge.

**Table I:** List of materials used for the set-up of closed tube irrigation system

Materials	Quantity
14 gauge (orange) cannula	2
Drip line	2
Sodium chloride 0.9% irrigation	
solution in 3 L bag	1 per day
Three-way stopcock	1
Urine drainage bag	1
Disposable dressing set and antiseptics	1
2% lignocaine	As required
Transparent adhesive film	
(to secure cannulas)	2
Drip stand	1
Sterile gloves, mask and apron	1

## Discussion

Simple closed irrigation-drainage of septic knee in adults has been described in the literature as early as 1980s^4,5^. This technique was initially used in the early stage of joint infection instead of repeated needle aspirations^4,5^. It did not gain much popularity possibly because of the introduction of effective arthroscopic management of knee septic arthritis in the 1980s. However, improvised suction-irrigation system is still occasionally used as an adjunct post arthroscopic procedures in late stages septic arthritis^2^.

In any irrigation-drainage system, there is a concern of possible “highway effect” in which the irrigation fluid takes the path of least resistance and flows through the joint without reaching all the knee joint compartments^2,3^. We therefore incorporated a daily thirty minutes cyclical distension-irrigation process. Outflow tube was stopped for a few minutes, leaving the inflow system going, then outflow tube was released and the process was repeated. At other times, a continuous irrigation and gravity drainage system was employed. We used 14 gauge cannulas which are large enough to allow abundant lavage and gravity drainage yet small enough to allow immediate closure on removal without the need for stitching as compared to drain tubes. We had one episode of blockage of the tubing by debris, which was easily managed with saline flush and aspiration.

This method could be a viable option in patients who are medically unfit to undergo surgery. The intention is to provide immediate joint decompression, reduction if not total elimination of the causative organisms and dilution of associated proteolytic and lysosomal enzymes responsible for cartilage damage. Without prompt treatment, the increasing pressure in an infected joint can lead to spontaneous joint perforation and the development of chronic fistula^3^. Chronic joint infection can also lead to osteomyelitis, joint stiffness and ankylosis^3^.

We kept the system for four days without complications. However, it is inadvisable to persist if there are signs of leakage into the soft tissue envelope due to the danger of compartment syndrome and cellulitis. It has to be kept in mind that complete lavage and joint assessment for debridement and synovectomy are only possible with open or arthroscopic procedures. Bedside irrigation-drainage is only an interim measure used to provide immediate joint decompression and reduce bacterial load but removal of fibrin coatings and necrotic tissues are not possible with this method. Therefore, to prevent recurrence, it is reasonable to proceed with formal surgical washout once the patient is surgically fit.

This is a simple, minimally invasive closed tube irrigation system that can be accomplished with commonly available materials in any general clinical setting for the initial treatment of septic knee in patients with complex medical problems who are unfit for surgery.
